# Supplementary material for: Heparin-binding motif mutations of human diamine oxidase allow the development of a first-in-class histamine-degrading biopharmaceutical
Source: eLife. 2021 Sep 3;10:e68542. doi: 10.7554/eLife.68542 (PMC8445614; doi:10.7554/eLife.68542)
Supplement: Figure 2—source data 1. [file elife-68542-fig2-data1.docx]

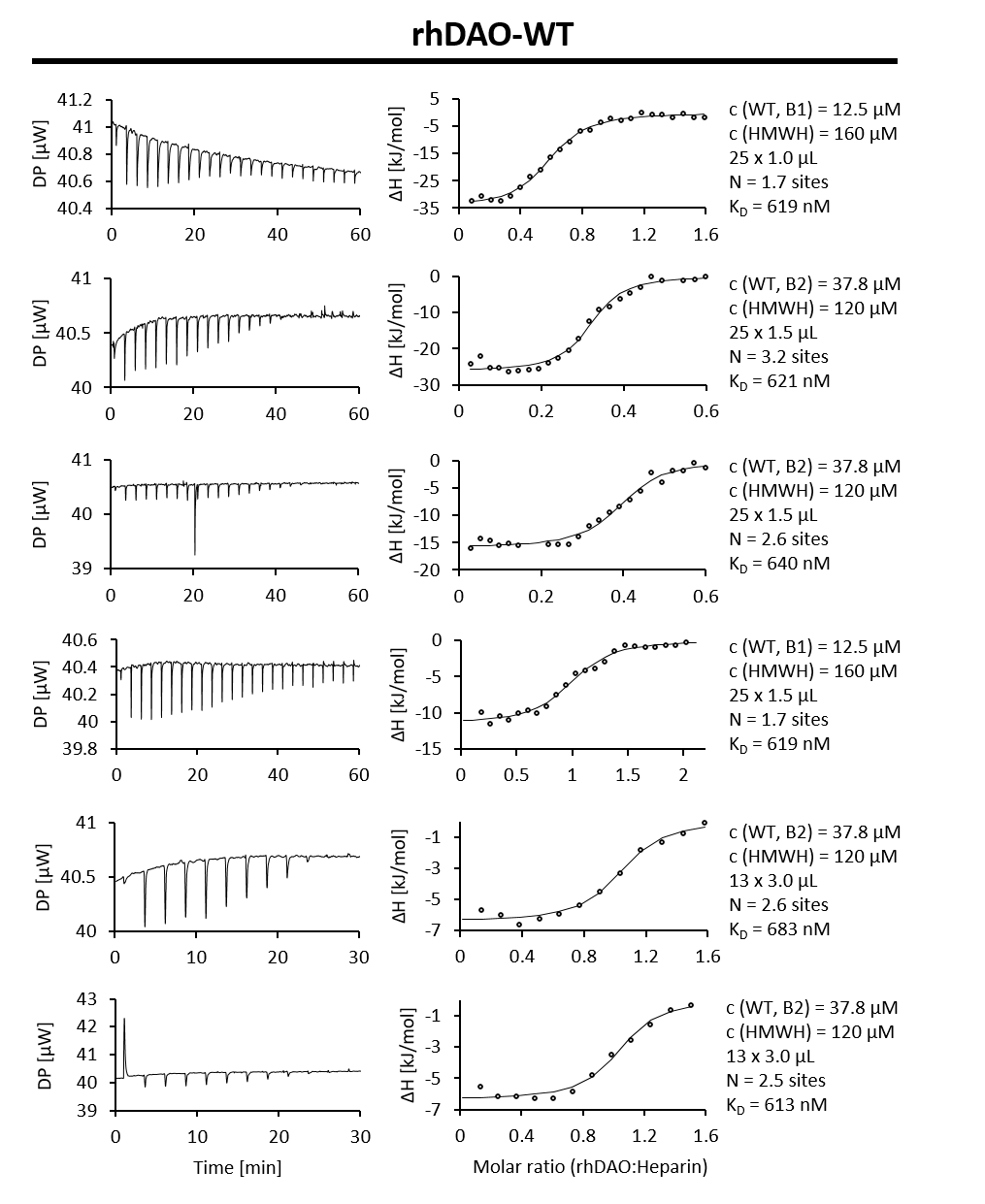


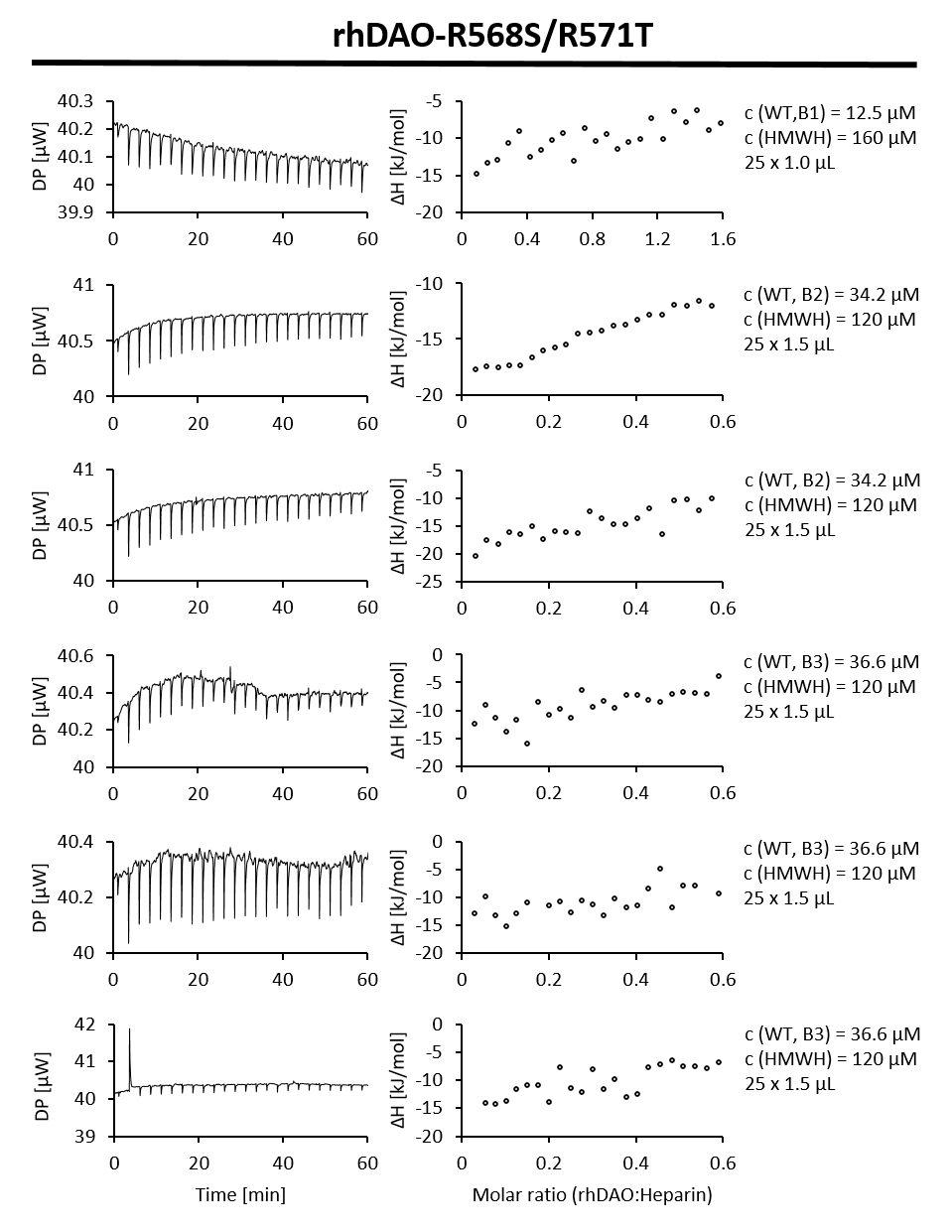


Figure 2 – source data 1. Raw plots and integrated heat plots of isothermal titration calorimetry of rhDAO-WT and rhDAO-R568S/R571T heparin-binding motif mutant with high-molecular weight heparin.

High-molecular weight heparin (HMWH) was titrated into rhDAO-R568S/R571T and rhDAO-WT from individual batches (B1, B2, B3) in 50 mM Hepes buffer with 150 mM KCl, pH 7.5. Protein and ligand concentrations, injection modes, resulting K_D_ values and number of binding sites (N) are shown. The six measurements of rhDAO-WT resulted in a mean (SD) K_D_ value of 633 (26) nM and a stoichiometry of 2.4 (0.6) DAO molecules binding to one HMWH molecule. 1 µM DAO corresponds to 170 µg/mL and 1 µM HMWH corresponds to 15 µg/mL or 3 units/mL. The wildtype data were recently published *(17)*.
