# Supplementary material for: Heparin-binding motif mutations of human diamine oxidase allow the development of a first-in-class histamine-degrading biopharmaceutical
Source: eLife. 2021 Sep 3;10:e68542. doi: 10.7554/eLife.68542 (PMC8445614; doi:10.7554/eLife.68542)
Supplement: Figure 3—source data 1. [file elife-68542-fig3-data1.docx]

**Figure 3 – source data 1. Statistical evaluation of flow cytometry data using various cell lines incubated with rhDAO-WT and rhDAO-R568S/R571T heparin-binding motif mutant.**

| Cell line | Normality by Shapiro-Wilk test | Equality of Variances | NC vs. R568S/R571T | NC vs.  WT | R568S/R571T vs. WT |
| --- | --- | --- | --- | --- | --- |
| **CHO-K1^*^** | **Yes** | **No** | **p < 0.05** | | |
| **HDF^**^** | **Yes** | **Yes** |  |  |  |
| **HDMVEC^*^** | **Yes** | **No** |  |  |  |
| **HeLa^*^** | **Yes** | **No** |  |  |  |
| **HepG2^*^** | **Yes** | **No** |  |  |  |
| **HUVEC^*^** | **Yes** | **No** |  |  |  |
| **LHCN-M2^*^** | **Yes** | **No** |  |  |  |
| **PODO^*^** | **Yes** | **No** |  |  |  |
| **SK-Hep1^*^** | **Yes** | **No** |  |  |  |

The cells were incubated with Alexa488-labeled rhDAO-WT and rhDAO-R568S/R571T (no DAO added = negative control) and 500 cells per sample were analyzed flow cytometrically (n = 4 biological replicates, 2 individual experiments in duplicate). The significance of differences in fluorescence intensities (median) of the negative control (NC), rhDAO-WT and rhDAO-R568S/R571T was tested. All comparisons were statistically significant with p-values < 0.05.

^*^ = Welch’s-ANOVA and Games-Howell test; ^**^ = ANOVA and Tukey’s HSD test
