# Supplementary material for: Heparin-binding motif mutations of human diamine oxidase allow the development of a first-in-class histamine-degrading biopharmaceutical
Source: eLife. 2021 Sep 3;10:e68542. doi: 10.7554/eLife.68542 (PMC8445614; doi:10.7554/eLife.68542)
Supplement: Figure 3—source data 2. [file elife-68542-fig3-data2.docx]

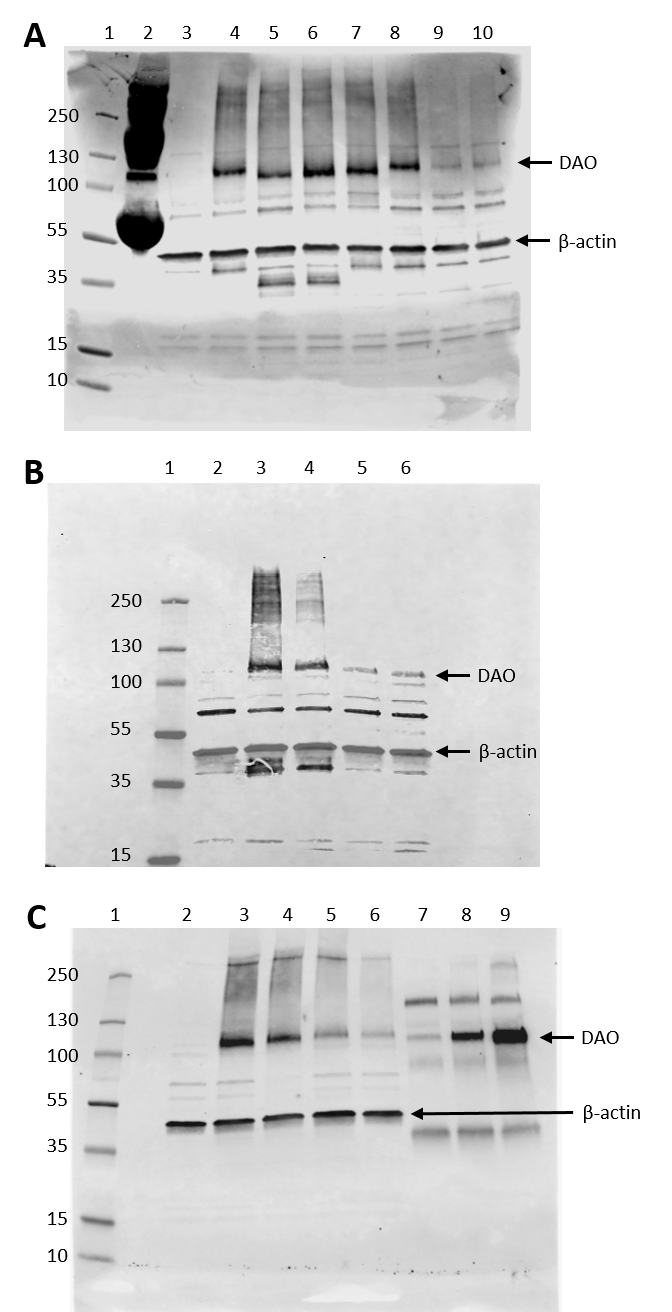


**Figure 3 – source data 1. Western blot raw data.**

**(A) SK-Hep1.** 1=Page Ruler Plus standard (kDa); 2=2 ng purified rhDAO-WT; 3=negative control; 4=rhDAO-WT; 5-7=rhDAO variants not relevant for this publication; 8=rhDAO-R568S; 9=rhDAO-R568S/R571T; 10=rhDAO-R568S/K575T. **(B) SK-Hep1.** 1=Page Ruler Plus standard (kDa); 2=negative control; 3=rhDAO-WT; 4=rhDAO-R568S; 5=rhDAO-R568S/R571T; 6=rhDAO-R568S/K575T. **(C) HUVEC/TERT2.** 1=Page Ruler Plus standard (kDa); 2=negative control; 3=rhDAO-WT; 4=rhDAO-R568S; 5=rhDAO-R568S/R571T; 6=rhDAO-R568S/K575T; 7=1 ng purified rhDAO-WT; 8=5 ng purified rhDAO-WT; 9= 20 ng purified rhDAO-WT.
