# Supplementary material for: Heparin-binding motif mutations of human diamine oxidase allow the development of a first-in-class histamine-degrading biopharmaceutical
Source: eLife. 2021 Sep 3;10:e68542. doi: 10.7554/eLife.68542 (PMC8445614; doi:10.7554/eLife.68542)
Supplement: Supplementary file 1. [file elife-68542-supp1.docx]

Supplementary file 1. The DAO heparin-binding motif is evolutionarily highly conserved in old world monkeys, great apes and humans.

|  | **568** | 569 | **570** | **571** | **572** | 573 | 574 | **575** |  |
| --- | --- | --- | --- | --- | --- | --- | --- | --- | --- |
|  | **R** | F | **K** | **R** | **K** | L | P | **K** |  |
|  | % sequence identity | | | | | | | | Mean |
| **Mammalia** | 89 | 98 | 21 | 49 | 21 | 99 | 100 | 67 | 68 |
| **OWM, GA, H** | 100 | 100 | 100 | 100 | 100 | 100 | 100 | 20 | 90 |
| **Others** | 86 | 97 | 4 | 39 | 4 | 99 | 100 | 76 | 63 |

Sequence alignment of 87 DAO mammalian species was performed using BLAST; 15 sequences from old world monkeys (OWM), great apes (GA) and humans (H) were analyzed separately; Others = *Mammalia* minus OWM, GA, H sequences. Positively charged residues are indicated in bold and underlined.
